# Neuronal apoptosis and inflammatory responses in the central nervous system of a rabbit treated with Shiga toxin-2

**DOI:** 10.1186/1742-2094-5-11

**Published:** 2008-03-21

**Authors:** Kiyomi Takahashi, Nobuaki Funata, Fusahiro Ikuta, Shigehiro Sato

**Affiliations:** 1 Department of Microbiology, Iwate Medical University School of Medicine, 19-1 Uchimaru, Morioka, Iwate 020-8505, Japan; 2 Department of Pathology, Tokyo Metropolitan Komagome Hospital, 3-18-22 Honkomagome, Bunkyo-ku, Tokyo 113-8677, Japan; 3 Niigata Neurosurgical Hospital and Brain Research Center, 3057 Yamada, Niigata, Niigata 950-1101, Japan

## Abstract

**Background:**

Shiga toxins (Stxs) are the major agents responsible for hemorrhagic colitis and hemolytic-uremic syndrome (HUS) during infections caused by Stx-producing *Escherichia coli *(STEC) such as serotype O157:H7. Central nervous system (CNS) involvement is an important determinant of mortality in diarrhea associated-HUS. It has been suggested that vascular endothelial injuries caused by Stxs play a crucial role in the development of the disease. The current study investigates the relationship between the cytotoxic effects of Stxs and inflammatory responses in a rabbit brain treated with Stx2.

**Methods:**

In a rabbit model treated with purified Stx2 or PBS(-), we examined the expression of the Stx receptor globotriaosylceramide (Gb3)/CD77 in the CNS and microglial activation using immunohistochemistry. The relationship between inflammatory responses and neuronal cell death was analyzed by the following methods: real time quantitative reverse transcriptase (RT)-polymerase chain reaction (PCR) to determine the expression levels of pro-inflammatory cytokines, and the terminal deoxynucleotidyl transferase (TdT)-mediated dUTP nick-end labeling (TUNEL) method to detect apoptotic changes.

**Results:**

Gb3/CD77 expression was detected in endothelial cells but not in neurons or glial cells. In the spinal cord gray matter, significant levels of Gb3/CD77 expression were observed. Severe endothelial injury and microvascular thrombosis resulted in extensive necrotic infarction, which led to acute neuronal damage. Conversely, in the brain, Stx receptor expression was much lower. The observed neuropathology was less severe. However, neuronal apoptosis was observed at the onset of neurological symptoms, and the number of apoptotic cells significantly increased in the brain at a later stage, several days after onset. Microglial activation was observed, and tumor necrosis factor (TNF)-α and interleukin (IL)-1β mRNA in the CNS parenchyma was significantly up-regulated. There was significant overexpression of TNF-α transcripts in the brain.

**Conclusion:**

This study indicates that Stx2 may not directly damage neural cells, but rather inflammatory responses occur in the brain parenchyma in response to primary injury by Stx2 in vascular endothelial cells expressing Gb3/CD77. These findings suggest that neuroinflammation may play a critical role in neurodegenerative processes during STEC infection and that anti-inflammatory intervention may have therapeutic potential.

## Background

Shiga toxin (Stx)-producing *Escherichia coli *(STEC), such as serotype O157:H7, produces 1 or both of the 2 antigenically distinct toxins Stx1 and Stx2 [1]. These toxins in the circulation of patients infected with STEC are the major cause of hemorrhagic colitis and the hemolytic-uremic syndrome (HUS) associated with the prodrome of diarrhea [2]. The extent and severity of extrarenal involvement, particularly the neurological impairment, are important determinants in the poor prognosis of STEC-associated HUS patients [3]. Pathological examination has shown that endothelial injury of small vessels and microvascular thrombosis are the prominent changes occurring in multiple organs, including the CNS [2,3]. Animal models treated with Stx1 [4,5] or Stx2 [6], also develop neurological symptoms and exhibit similar neuropathology. Stx1- or Stx2-binding was immunohistochemically demonstrated in small vessels and/or the subsets of some cells in the lesions, but not in the neurons [4,7].

Stx1 and Stx2 are AB_5 _holotoxins that consist of an enzymatic A subunit and 5 copies of a binding B subunit [8]. The B subunits specifically bind to the terminal [Galα (1–4)Gal] disaccharides of the receptor Gb3/CD77 consisting of a trisaccharide β-linked ceramide (globotriaosylceramide) that is expressed on the cell membrane [9,10]. Stxs are then internalized with Gb3/CD77 and retrogradely transported from the endosomes through the *trans*-golgi network and golgi apparatus to the endoplasmic reticulum [9]. The A subunit of the toxin is then cleaved and translocated into the cytosol; it then inhibits host cell protein synthesis by RNA *N*-glucosidase activity, resulting in cell death [10]. Recent studies have reported that cell death occurs by apoptosis [11,12]. Toxin-binding to Gb3/CD77 is the primary determinant of the cytotoxic and pathological effects of Stxs. However, Gb3/CD77 expression in the CNS parenchyma has not been well examined, except in the dorsal root ganglia (DRG) [13]. Furthermore, the direct cytotoxic actions of Stxs against neurons and glial cells remain unclear.

CNS responses to a variety of insults incorporate a wide spectrum of cellular responses that include microglial activation [14,15] and local production of endogenous molecules such as pro-inflammatory cytokines, reactive oxygen, and nitrogen species that affect neuronal integrity [reviewed in [16] and [17]]. These inflammatory responses in the CNS are now recognized to play a critical role in the pathogenesis and progression of acute and chronic neurodegenerative diseases, including stroke [18], Alzheimer's disease [19,20], Parkinson's disease [21], and viral infection [22,23]. It has been demonstrated that pro-inflammatory cytokines, particularly TNF-α, induce neuronal apoptosis in human brain cell cultures and animal models through TNF/TNF receptor signaling [24,25] and further production of other inflammatory and/or neurotoxic molecules [19,21]. Administration of TNF-α to mice infected with *Escherichia coli *O157:H7 modified the neurological signs and pathology [26]. In the case of neurological impairment during STEC infection, however, little is known about the relationship between inflammatory responses in the CNS due to the toxin and the neurodegenerative events in either patients or animal models.

In the current study, we showed the regional distribution and cellular localization of Gb3/CD77 expression in the CNS parenchyma of a rabbit model in order to examine whether the toxins are able to directly injure neuronal cells. Further, we investigated the relationship between neuropathology and the interaction of toxin with Gb3/CD77 and that between inflammatory processes in the CNS and neuronal cell death in rabbits treated with Stx2.

## Materials and methods

### Toxin and animals

The Stx2 preparation was kindly provided as described previously [27] by Denka Seiken Co., Ltd., (Niigata, Japan). The same lot of toxin was used for all experiments in this study. The protein content of the toxin was 130 μg/ml as determined by Bradford method. The endotoxin content was 34.5 pg/ml as measured by a limulus test (ES-Test Wako Kit), using computerized turbidimetric equipment (Wako Chemical Industries, Co. Ltd, Osaka, Japan) [28]. Biological activity of Stx2 assessed by cytotoxicity on Vero cells was found to be 5 × 10^6 ^CD_50_/μg protein.

Japanese male white rabbits (Japan SLC, Inc., Hamamatsu, Japan) weighing 2.2 to 2.4 kg were used throughout the experiments. All protocols used in this study were in accordance with the Guidelines for Animal Experiments of Iwate Medical University. Animals were acclimated to standard laboratory conditions with free access to rabbit chow and water. In each experiment, at least 2 rabbits per group were used. The toxin was injected into the left marginal ear vein in a single bolus. Six doses of 0.1 to 4.0 μg/kg body weight of Stx2 were administered for clinical evaluation. For other experiments, including histopathological examination and gene expression, 2.5 μg/kg of toxin were administered by the same route. Controls were untreated rabbits injected with PBS(-) as above.

### Laboratory tests

Blood samples were collected before administration of Stx2 and at the onset of paralysis. Hematology, blood urea nitrogen, creatinine, alanine aminotransferase, aspartate aminotransferase, and amylase were measured by standard laboratory procedures in our hospital. The presence of blood in stools was tested using the Occult Blood Slide 5 Kit (Shionogi & Co., Ltd., Osaka, Japan).

### Tissue preparation

Animals were intravenously injected with a lethal dose of pentobarbital sodium and then rapidly perfused with sterile physiological saline transcardially, followed by 2% paraformaldehyde (PFA). Brain that denote the cerebrum, midbrain, cerebellum and brain stem, spinal cord with root ganglia and nerve fibers were removed and post-fixed with 4% PFA overnight at 4°C. The tissue was paraffin-embedded and sliced at 5 μm for histopathology, immunohistochemistry, and the terminal deoxynucleotidyl transferase (TdT)-mediated dUTP nick end-labeling (TUNEL) assay. For the immunofluorescence assay (IFA) tissue blocks were freshly embedded and frozen after cryo-protection as described previously [29]. Tissue blocks were stored at -70°C until sectioning.

### Immunohistochemistry

Localization of Gb3/CD77 was examined by IFA. Cryosections cut at 7 μm were fixed with 4% PFA for 10 min immediately prior to IFA and incubated with anti-human CD77 rat IgM antibody [13] (1:100 dilution, Beckman Coulter, Inc., Fullerton, CA) or with rat IgM isotype as a negative control (1:100 dilution, Beckman Coulter) for 24~96 hrs at 4°C, followed by reaction with anti-rat IgM goat IgG F(ab)_2 _antibody-FITC conjugate (1:200 dilution, Beckman Coulter) for 30 min at room temperature. To examine the cellular localization of Gb3/CD77, double-staining with rhodamine conjugated *Ricinus communis *agglutinin I (RCA 120) (1:5,000 dilution, Vector Laboratories, Inc., Burlingame, CA) was carried out overnight at 4°C on the same sections after Gb3/CD77 detection. The sections were observed with the LSM 510 confocal laser scanning microscopy (Carl Zeiss, Oberkochen, Germany).

Paraffin-embedded sections were stained for microglial cells as described previously [23,30], with some modification. Briefly, deparaffinized sections were pretreated with 3% H_2_O_2 _for 10 min to quench the endogenous peroxidase activity, and were then incubated with biotinylated RCA 120 (1:3,000 dilution) or *Griffonia simplicifolia *lectin I-B4 isolectin (GSL I-B4) (1:2,000 dilution, Vector) overnight at 4°C. This was followed by a reaction with peroxidase conjugated streptavidin (DAKO). The color reaction was performed using 0.5 mg/ml of diaminobenzidine (DAB) (Sigma-Aldrich, Inc., St. Louis, MO) substrate solution with 0.02 % of H_2_O_2_. Sections were counterstained with methyl green and mounted.

### TUNEL assay

In order to detect *in situ *DNA fragmentation, the TUNEL assay was performed using the *In situ *Apoptosis Detection Kit (Takara Bio Inc., Otsu, Japan) according to the manufacturer's protocols. Briefly, deparaffinized sections were permeabilized with proteinase K (DAKO) and treated with 3% H_2_O_2 _as above. Sections were incubated with Labeling Safe Buffer, i.e, an end-labeling mixture containing FITC-conjugated dUTP and TdT Enzyme, at 37°C for 60 min. Tissue sections were reacted with anti-FITC horseradish peroxidase-conjugated antibody for 30 min at 37°C. Color reaction with DAB was performed, followed by counterstaining with methyl green as above. TUNEL assay controls included omission of TdT and sections from rabbits (n = 2) treated with PBS (-). To estimate apoptotic cell death quantitatively, tissue sections of rabbits treated with 2.5 μg/kg of Stx2 were examined at the onset and at a later stage several days after onset (n = 2 in each group). Apoptotic cells in 16 to 50 fields and TUNEL-positive vessels in 140 to 160 fields were counted at 100× magnification in 3 sections from each region of the brain parenchyma.

### Real-time quantitative reverse transcription-polymerase chain reaction

Total RNA was extracted from 12 regions of the CNS using NucleoSpin RNA II (Macherey-Nagel GmbH & Co. KG, Düren, Germany) and 1 μg of total RNA was used for cDNA synthesis with oligo dT and MMLV-RT (Invitrogen Corp., Carlsbad, CA). Two μl of each cDNA product was subjected to TNF-α and IL-1β mRNA quantitation using SYBR Green PCR Master Mix [31] and an ABI PRISM 7700 Sequence Detection System (SDS) (Applied Biosystems, Foster City, CA), followed by a dissociation curve analysis and subsequent agarose gel electrophoresis to confirm amplification specificity. The following primer sets were used: TNF-α (size, 243 bp; melting temperature (Tm), 87.5°C), forward [32]: 5'-AGCCCACGTAGTAGCAAACCC-3', reverse: 5'-GAGAGGAGGTTGACCTTGTT-3' [GenBank: M12485 and M12486]; IL-1β (size, 264 bp; Tm, 81.0°C), forward [33]: 5'-GAATCTGAACCAACAAGTGG-3', reverse: 5'-ATGTACCAGTTGGGGAACT-3' [GenBank: M26295]; and GAPDH was used as an endogenous reference (size, 308 bp; Tm, 88.3°C), forward: 5'-TCTCTCAAGATTGTCAGCAA-3', reverse: 5'-AGGTCCACGACCGACACGTT-3' [GenBank: L23961]. All experiments were carried out twice in triplicate, amplification plots were analyzed using the ABI Prism 7700 SDS version 1.7 software. Expression levels of mRNA were quantified by the relative standard curve method, according to the User Bulletin #2 for ABI Prism 7700 SDS. Briefly, the target amount was divided by the endogenous reference (GAPDH) amount to obtain a normalized target value for each sample. The relative quantity of cytokine mRNA levels was calculated by dividing each of the normalized target values by the normalized calibrator value for each sample, and was expressed as *n*-fold difference and mean ± SD. CNS samples from control rabbits treated with PBS(-) were used as calibrators in this study.

### Statistics

Mean ± SEM or mean ± SD were determined, and to compare mean values in 2 separate groups, Student's *t*-test was used. Values of *P *< 0.05 were considered to be significant.

## Results

### Clinical findings and laboratory studies

In all rabbits injected with 0.1 to 4.0 μg/kg of Stx2, digestive symptoms such as anorexia, weight loss, and diarrhea were observed (Table 1). The 50% lethal dose was 0.79 μg/kg, and neurological symptoms developed in 2 of 4 rabbits (50%) injected with 1.0 μg/kg of Stx2 and in 5 of 6 (83.3%) treated with 2.0 to 4.0 μg/kg of toxin (Table 1). As early as 28~42 h after injection, among the 23 animals injected with 1.0 to 4.0 μg/kg (including the 16 in table 1), 18 showed neurological symptoms, which included mild gait disturbance in 2 animals and paralysis in 16 rabbits. There were no significant changes in the hematological and blood biochemical values at the onset of neurological symptoms when compared with those obtained before administration of the toxin (not shown), except for a slight elevation in the leukocyte count (8345 ± 394 and 9602 ± 755, before administration and at the onset of neurological symptoms, respectively).

**Table 1:** Dosage range of Stx2 and clinical findings

Dose (μg/kg)	neurological symptoms (%)	Diarrhea/occult blood	Body weight loss (%)
0.1	1^a ^/5 (20)	-~+/-~+	8.0~38.8
0.25	0/4 (0)	+/+	13.2~25.6
0.5	0/4 (0)	+/+	22.0~36.7
1.0	2/4 (50)	+/+	26.7
2.0	5/6 (83.3)	+/+	-^b^
4.0	5/6 (83.3)	+/+	-^b^

^a^Transient gait disturbance was observed on the 4th to 6th day after administration of Stx2.

^b^not measured.

### Gb3/CD77 expression and histopathological evaluation

#### Regional distribution and cellular localization of Gb3/CD77

**Figure 1:**
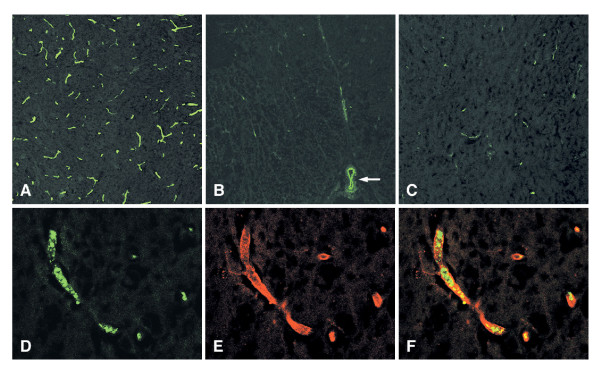
**Immunofluorescence analysis of the localization of Stx receptor Gb3/CD77 in the rabbit CNS**. Intense fluorescence was detected on the blood vessels of the spinal cord gray matter **(A)**, whereas fluorescent signals were much weaker on those of the spinal cord white matter except anterior spinal artery (arrow) **(B) **and hippocampus **(C)**. Confocal images of double staining with rhodamine conjugated RCA 120 showed the different fluorescence signal patterns from Gb3/CD77 and revealed the colocalization of both in the small vessels and capillaries; Gb3/CD77 **(D)**, RCA 120 **(E), **and merged **(F)**. (Original magnification: **(A)**-**(C) **200×; **(A)**-**(E) **630×)

Specific and intense fluorescence signals were strongly detected on the blood vessels of the spinal cord gray matter (Fig. 1A). Whereas the intensity of fluorescence was weaker in the white matter of the spinal cord (Fig. 1B), root ganglia (not shown), and brain parenchyma (Fig. 1C). Signals were negative in the control sections incubated with rat IgM isotype antibody (not shown). Double staining with rhodamine-conjugated RCA 120, which recognizes and specifically binds to terminal [D-Galβ (1–4)-D-GlcNAc] disaccharides [34], demonstrated different fluorescence signal patterns from Gb3/CD77 and revealed colocalization of both in the small vessels and capillaries (Fig. 1D to 1F); however, this was not evident in other subsets of cells in the CNS parenchyma.

#### Spinal cord

**Figure 2:**
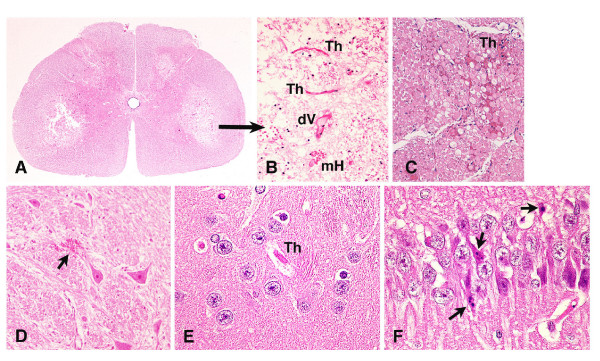
**Neuropathology in the rabbits treated with 2.5 μg/kg Stx2 (H&E staining)**. **(A) **The lower segment of the lumbar spinal cord showed extensive necrotic infarction in the gray matter at the onset of severe paraplegia. **(B) **Higher magnification of ischemic lesions in the panel **(A)**, small vessels with fibrin-like thrombi (Th), degenerated vessels (dV), and microscopic hemorrhage (mH) were observed. **(C) **Nerve roots of the rabbit with ataxic gate showed microvascular thrombi and microscopic hemorrhage. Some nerve fibers were swollen and/or degenerated. In contrast to the spinal cord, **(D) **microscopic hemorrhage (arrows) were scattered in the brain parenchyma. At a later stage, neurons in the basal ganglia showed atrophy, darkly stained cytoplasm **(E)**, and in the hippocampus some pyramidal neurons morphologically showed apoptotic changes with shrunken cytoplasm and apoptotic bodies (arrows) **(F)**. (Original magnification: **(A) **5.2×; **(B) **250×, **(C) **and **(D) **100×, **(E) **and **(F) **400×.)

The prominent pathological damage was massive necrotic infarction in irregular form, which was mostly noted in the gray matter of the cervical and lower thoracic to lumbar cords (Fig. 2A). In and around the ischemic lesions, neuronal bodies were damaged and axons were swollen. Small vessels and capillaries were frequently injured and occluded by fibrin-like thrombi (Th in Fig. 2B) and some exhibited eosinophilic exudates (dV) or microscopic hemorrhage (mH) as shown in Fig. 2B. In a rabbit with mild ataxic gate but not paralysis, thrombi and/or microscopic hemorrhage were only observed in the nerve roots. Some axons were swollen and some of these were degenerated or lost (Fig. 2C), neurons in the DRG exhibited normal appearance (not shown).

#### Brain parenchyma

In contrast to the severe ischemic damages in the spinal cord, a few foci of microscopic hemorrhage (Fig. 2D) and/or ischemic lesions were scattered in the brain parenchyma and fibrin-like thrombi were often observed (Th) in small vessels and capillaries at the onset of symptoms. Neurons were damaged in the basal ganglia (Fig. 2E) and thalamus at onset. These cells showed atrophy and darkly stained cytoplasm. Several days after onset, at a later stage, some neurons in CA1 of the rabbit hippocampus morphologically exhibited an apoptotic appearance such as condensed and fragmented nuclei in shrunken cytoplasm (Fig. 2F).

#### Vascular damages

**Figure 3:**
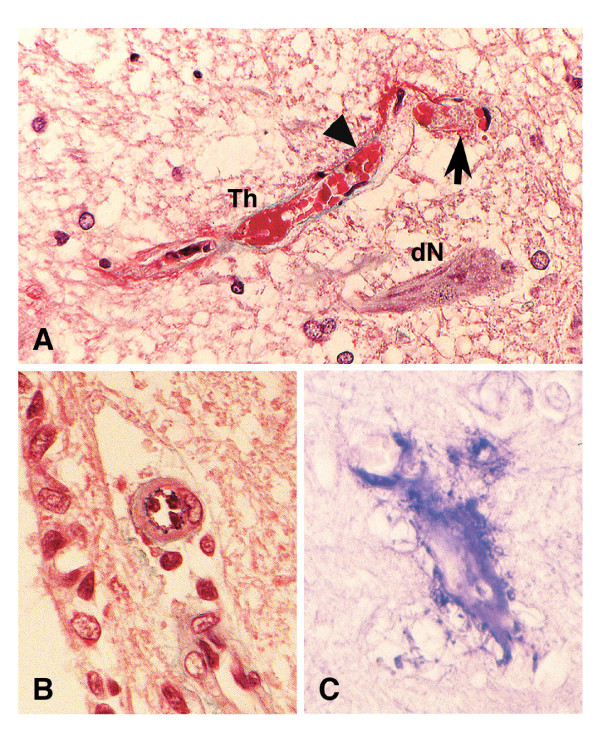
**Vascular degeneration in the rabbits treated with 2.5 μg/kg Stx2**. **(A) **In the infarction lesions in spinal cord gray matter, small vessels were damaged with extravascular exudates and occluded with fibrin-like (Th) and platelet (arrow) thrombi and with fragmented erythrocytes (arrow head). Some arteries and arterioles **(B) **in the spinal cord showed an endothelium with pycnotic nuclei and swollen cytoplasm as well as thickening of the vessel walls. **(C) **PTAH staining indicated that most of the thrombi and exudates or precipitates around the capillaries were formed with fibrin in both the spinal cord and brain parenchyma. **(A) **&**(B)**: Elastica-Goldner staining. (Original magnification: 400×)

Elastica-Goldner staining showed that small vessels in the infarction lesions in the spinal cord gray matter were mainly damaged (Fig. 3A) and occluded by fibrin-like thrombi (Th). Platelet thrombi (arrow in Fig. 3A) or fragmented red blood cells (arrow head in Fig. 3A) were also present in a few vessels. Arterioles in the spinal cord showed endothelium with pycnotic nuclei and swollen cytoplasm as well as thickening of the vessel wall, but not formation of thrombi (Fig. 3B). These vascular damages were less severe in the brain parenchyma. Phosphotungstic acid hematoxylin (PTAH) staining revealed that most of the thrombi in small vessels and exudates or precipitates around capillaries were formed with fibrin in both the spinal cord (Fig. 3C) and brain parenchyma.

### Apoptotic cell death of neuronal cells and endothelial cells in the brain parenchyma

**Figure 4:**
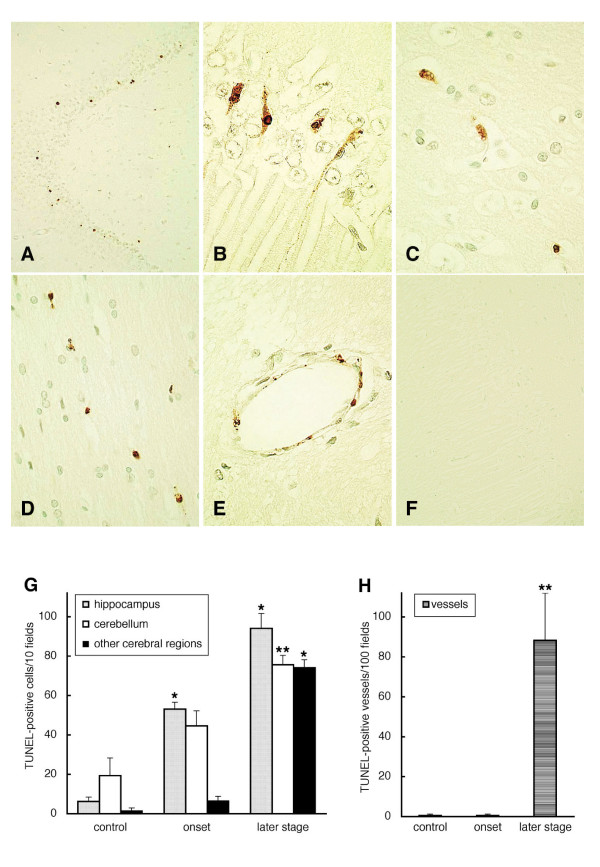
**TUNEL assay of brain sections from rabbits treated with 2.5 μg/kg of Stx2**. **(A) **Condensed dark brown cells were detected in the dentate gyrus of the hippocampus at the onset of paralysis. TUNEL-positive neurons showing shrunken cytoplasm, pycnotic nuclei, and/or apoptotic bodies increased at a later stage in the following other regions: CA1 of the hippocampus **(B) **and cerebral cortex **(C)**. Apoptotic glial cells in pons **(D) **and small vessels **(E) **with condensed cytoplasm and apoptotic bodies were also observed at a later stage. **(F) **TUNEL assay without TdT showed no positive staining. **(G) **Quantitative evaluation of TUNEL-positive cells per 10 fields in the hippocampus, cerebellum, and other cerebral regions, including the basal ganglia, thalamus, and cerebral cortex, and **(H) **TUNEL-positive vessels per 100 fields in the cerebral parenchyma at the onset of neurological symptoms and at a later stage when compared with control rabbits treated with PBS(-) (n = 2 in each group). The number of apoptotic cells significantly increased in the hippocampus at both onset and a later stage, and in granular layers of the cerebellum and other cerebral regions at a late stage. TUNEL-positive vessels were significantly elevated at a later stage. *: *P *< 0.01 and **: *P *< 0.05. (Original magnification: **(A) **and **(F) **200×, **(B) **800×, **(C) **and **(E) **600×, **(D) **400×)

At the onset of paralysis, apoptotic granular neurons with condensed brown DAB precipitate were observed in the dentate gyrus of the hippocampus (Fig. 4A) and granular layers of the cerebellum. TUNEL-positive neurons with apoptotic bodies increased at a later stage several days after onset in the pyramidal neurons in CA1 of the hippocampus (Fig. 4B). Apoptotic changes were also observed diffusely in neurons in other cerebral regions, namely, the basal ganglia, thalamus and cerebral cortex (Fig. 4C), among glial cells in the pons (Fig. 4D), and in some endothelial cells of the small vessels and capillaries in the cerebrum (Fig. 4E). TUNEL assay with omission of TdT (Fig. 4F) was negative in all the regions. The number of apoptotic cells per 10 fields (Fig. 4G) and of TUNEL-positive vessels per 100 fields (Fig. 4H) at onset or at a later stage were compared with those of control rabbits treated with PBS(-). In the hippocampus, apoptotic neurons were significantly elevated at both onset (53.1 ± 3.49) and a later stage (94.0 ± 7.65), (*P *< 0.01). Apoptotic cells in the granular layers of the cerebellum (75.9 ± 4.76, *P *< 0.05), other cerebral regions, including the basal ganglia, thalamus and cerebral cortex (74.1 ± 4.04, *P *< 0.01), and TUNEL-positive vessels (88.2 ± 23.74, *P *< 0.05) were significantly increased at a later stage.

### Inflammatory responses in CNS

#### Microglial activation

**Figure 5:**
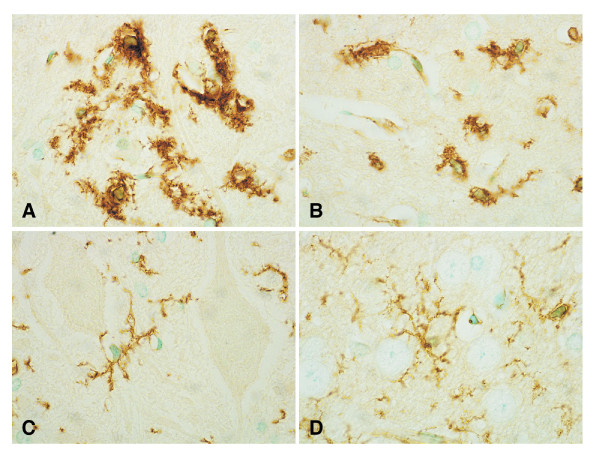
**GSL I-B4 isolectin staining for microglia**. In the rabbits treated with 2.5 μg/kg of Stx2, a large number of microglia exhibited an ameboid form in and around the ischemic lesions in the lumbar cord gray matter **(A) **and extensively increased in the brain parenchyma, thalamus **(B) **at the onset of paralysis, when compared with the lumbar cord **(C) **and thalamus **(D) **of control rabbits injected with PBS(-), showing a ramified form of microglia. (Original magnification: 800×)

At the onset of paralysis, intense GSL I-B4 staining of microglial cells were diffusely observed in both the spinal cord and brain. Microglial activation was obvious due to the morphological change in the cells. Activated microglia were markedly found in and around ischemic lesions in the spinal cord (Fig. 5A) and extensively increased in the brain parenchyma (Fig. 5B). A large number of microglia exhibited an ameboid form when compared with the ramified form in the control sections of the spinal cord and brain parenchyma (Fig. 5C and 5D, respectively). Interestingly, microglial activation greatly increased before the onset of neurological symptoms, as early as 6 to 12 h after Stx2 injection and was also observed at a later stage.

#### Up-regulation of pro-inflammatory cytokine mRNA

**Figure 6:**
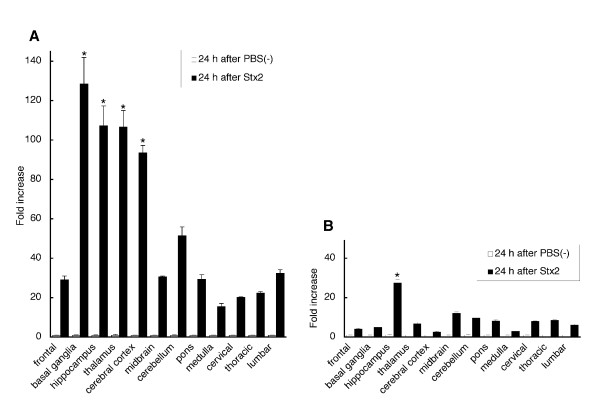
**Real-time quantitative PCR study**. TNF-α **(A) **and IL-1β **(B) **transcripts in 12 regions of the rabbit CNS at 24 h after injection of PBS(-) as a calibrator (open bar: 1 ± SD) or 2.5 μg/kg Stx2 (closed bar: mean ± SD). All data were normalized to the internal reference GAPDH amounts and expressed as an *n*-fold increase relative to normalized calibrator values in each region. Both TNF-α and IL-1β mRNA levels were greatly up-regulated. In particular, TNF-α transcripts were significantly overexpressed in the cerebrum, basal ganglia, hippocampus, thalamus, and cerebral cortex. *: *P *< 0.01 compared to frontal region.

Since microglial activation was observed before onset, the expression levels of pro-inflammatory cytokine mRNA were examined 24 h after administration of 2.5 μg/kg Stx2 (n = 2) in the frontal area, basal ganglia, hippocampus, thalamus, temporal cortex, midbrain, cerebellum, pons and medulla, cervical, thoracic and lumbar spinal cord, and compared to the expression levels in the same regions of rabbits injected with PBS(-) as a calibrator (n = 2). Expression levels of TNF-α (Fig. 6A) and IL-1β (Fig. 6B) mRNA increased in all regions of the rabbits treated with Stx2 although the neurological signs had not developed at this time point. TNF-α transcripts were substantially up-regulated (by 15.55 ± 1.66~128.53 ± 13.54 fold increase) and particularly overexpressed in 4 regions of the cerebrum, namely, the basal ganglia, hippocampus, thalamus, and cerebral cortex (128.53 ± 13.54, 107.35 ± 10.05, 106.73 ± 8.35, 93.65 ± 3.76 fold, respectively); TNF-α expression also greatly increased in the cerebellum (51.62 ± 4.43 fold). Whereas the IL-1β mRNA levels were moderately increased (by 2.63 ± 0.30~12.14 ± 0.68 fold), except in the hippocampus (27.60 ± 1.71 fold). Comparable results were obtained using different set of rabbits.

## Discussion

The present study demonstrated that in the brain of rabbits treated with purified Stx2, neurodegenerative events coincide with inflammatory responses characterized by microglial activation and production of pro-inflammatory cytokines. In addition, these events greatly differed from those in the spinal cord although the primary action of Stxs was cytotoxicity against vascular endothelial cells expressing Gb3/CD77, not against neuronal cells, in both the spinal cord and brain.

Early studies showed that neuropathology, such as endothelial injury with thrombi and ischemic damage in rabbits treated with Stx1 or Stx2, was severe and extensive in the spinal cord but minimal in the brain [4,6]; this indicated specific binding of Stx1 to the endothelial cells of capillaries and vascular damage in the affected rabbit tissue [4]. These are in accordance with our results that neuropathological damage is markedly associated with regional distribution and cellular localization of Gb3/CD77 expression in the CNS parenchyma, indicating that the cytotoxic effects of Stx2 on endothelial cells was proportionate to the extent of Gb3/CD77 expression. Subsequent progression of acute neuronal injury in the spinal cord gray matter is mainly responsible for the paralysis that is the prominent clinical manifestation in the rabbit model treated with toxins.

Unlike the severe and massive ischemic insults in the spinal cord, the remarkable histopathology observed in the brain was due to neuronal cell death as evaluated by the TUNEL assay. Neuronal apoptosis was observed in both the dentate gyrus of the hippocampus and granular layers of the cerebellum at the onset of neurological symptoms, thereafter, significantly increased at a later stage several days after onset in more cell subsets, such as the pyramidal neurons in the hippocampus, neurons in other regions, and glial cells in the white matter as well as in vascular endothelial cells in the cerebral parenchyma. Toxin binding was detected in ependymal cells and the myelin sheath as well as the endothelial cells in brain lesions of the rabbit injected with Stx2 [7], but not in the neurons [4,7]. Neuronal changes in a baboon exposed to a high level of Stx1 [35] and in a rabbit treated with Stx2 [36] were microscopically demonstrated. Further, Stx2 administered into the cerebroventricular space was detected in astrocyte and neuronal fibers in the rat corpus striatum, resulting in their ultrastructural alterations [37]. However, the mechanism by which toxin binding leads to neuronal damages has not been elucidated. Besides the brain endothelial cells, GB3/CD77 expression was not detected in either neurons or glial cells by the IFA. This suggests that Stxs might not directly exhibit essential cytotoxic action, i.e., protein synthesis inhibition, against neuronal cells. Accordingly, neuronal degeneration following Stx2 administration is very likely caused by indirect effects, rather than by direct cytotoxicity of the toxin against neuronal cells.

In the current study, immunoreactivity and morphological changes of microglia were increased as early as 12 h after Stx2 injection and TNF-α transcripts were markedly increased in the brain before onset of neurological symptoms. Most pathological changes in the CNS are accompanied by an involvement of glial cells, particularly microglia, which are activated at an early stage and change their morphology rapidly in response to even minor pathological conditions in the CNS [15,38]. We previously observed widespread activation of microglia in the brain parenchyma in acute fatal measles cases in which only endothelial cells of small vessels were infected with the measles virus [39]. This suggests that endothelial injury by Stx2 can also evoke microglial activation. Endogenous thrombin generated by vascular damage might contribute to activation of microglia [40]. Activated microglia, by inhibiting both the basal formation of new neurons and increased neurogenesis in response to brain insults, may contribute to the neurodegenerative processes [41]. Furthermore, activated microglia are known to produce pro-inflammatory cytokines, such as TNF-α and IL-1β [15,23,38,40], which are observed long before significant neuronal death at the early phases of neuroinflammatory events [16]. TNF-α is shown to induce neurodegeneration directly, through signaling death pathway of TNF-α/p55 TNF receptor-1 in neurons [24,42] and oligodendrocytes [25] or through inducing activation of caspase-3 in a mixed neuro-glial culture system [43], furthermore, indirectly, through inhibiting receptor signaling for neuronal survival via the protective peptide insulin-like growth factor I [44] or through inducing microglial glutamate release in an autocrine manner [45]. Collectively, our observations that inflammatory responses, such as microglial activation and TNF-α overexpression, occurred in the brain of the rabbits at an early stage suggest that neuronal apoptosis and degeneration is probably triggered by neuroinflammation that is induced in response to primary endothelial injury by Stx2. The synergistic effects of TNF-α and IL-1β, however, have been demonstrated in human fetal brain cell cultures [42], thus suggesting that IL-1β, which showed a small increase in our animal model, might also participate in the neurodegenerative processes.

Previous studies have demonstrated that Stx1 induces apoptosis in the brain microvascular endothelial cells [46] in a dose- and time-dependent manner [11,47]. Toxins inhibit the expression of the anti-apoptotic Bcl-2 family member Mcl-1 in endothelial cells [12], and caspase inhibitors block apoptotic cell death [11,46,47]. Gb3/CD77 expression on the membrane of these cells is required to activate the death signal cascade and enhance Bax expression [11]. Therefore, in contrast to neuronal cell death, endothelial apoptosis in the brain parenchyma is most likely Gb3/CD77-dependent and induced by the direct cytotoxic effects of Stx2. Moreover, *in vitro *studies have shown that pro-inflammatory cytokines such as TNF-α and IL-1β markedly increased the Gb3/CD77 content of and Stx-binding to brain microvascular endothelial cells, resulting in the up-regulation of cytotoxicity [48-50] and apoptotic cell death [46].

There are no specific therapies to ameliorate the course of neurological involvement during STEC infection. However, treatment with nafamostat mesilate (6-amidino-2-naphthyl *p*-guanidinobenzoate dimethanesulfonate) significantly decreased the pro-inflammatory cytokine levels in the brain and changed the neuropathology in gnotobiotic mice infected with STEC O157:H7 [26]. Inhibition of Stx1-induced TNF-α production with anisodamine (raceanisodamine hydrochloride) prolonged the survival time and decreased the lethality of mice injected with the toxin [51]. Suppressing neuroinflammatory responses generally [26,52] or neurotoxic molecules selectively [19,21,45] has recently been recognized as an effective therapeutic strategy for neurodegenerative diseases. Therefore, anti-inflammatory compounds or selective inhibitors may have therapeutic potential for Stx-induced neurological manifestation as well.

## Conclusion

The Stx receptor Gb3/CD77 was expressed on endothelial cells in the brain parenchyma, but not on neurons or glial cells. Therefore, vascular damages and induction of endothelial apoptosis were very likely caused by direct cytotoxic action of Stx2. Apoptotic cell death of neurons and/or glial cells, however, may result from inflammatory responses in CNS following primary endothelial injury by Stx2; this is because microglial activation and significant up-regulation of TNF-α and IL-1β transcripts occurs in the brain parenchyma of rabbits treated with Stx2. Collectively, these results suggest that inflammatory responses play a critical role in progression of neurological impairment during STEC infection; however, the expression of Gb3/CD77 in the human CNS still remains unclear.

## Competing interests

The author(s) declare that they have no competing interests.

## Authors' contributions

KT designed the experiments and carried out most of the lab work. AF and FI performed the histopathological evaluation, including a part of tissue staining. SS aided in quantitative and statistical analysis and edited the manuscript.
